# Integrated disease management, optimal effort allocation between prevention and control

**DOI:** 10.1038/s41598-025-16318-5

**Published:** 2025-08-18

**Authors:** Mathieu Bonneau, Nathalie Mandonnet, Carine Marie-Magdeleine, Harry Archimède, Jean-Christophe Bambou

**Affiliations:** ASSET UR0143, INRA, 97170 Guadeloupe, Petit-Bourg France

**Keywords:** Ecological modelling, Agroecology

## Abstract

Prevention and control are two complementary strategies for disease management. In the absence of disease, prevention could be viewed as wasteful. On the contrary, control requires efforts, only in the presence of the disease. But in some cases, control strategies are implemented a long time after the disease started, when irrevocable damages could be already observed. Deciding how much efforts are dedicated to prevention and control is a difficult problem in practice. In this work, we formulated a simple mathematical model to balance the trade-off between prevention and control efforts, with the expected loss due to disease. We derived an analytical form of the optimal prevention and control efforts and showed that the amount of control and prevention efforts depends on the relative values of the control and prevention efficacy, weighted by the probability of disease occurrence. We illustrated our framework for the management of gastro-intestinal nematodes in small-ruminants. In particular to discuss the use of different common management methods, such as systematic or targeted anthelmintic treatments, and mixed grazing. We proposed simple decision rules that could help designing future management practice and research efforts in this area. For examples, we computed the minimal anthelmintic efficacy for treatment to be economically viable. We also discussed the optimal cost and efficacy of a monitoring method for implementing targeted selective treatment, or the maximal cost of mixed grazing implementation. This framework is particularly useful to discuss the cost of new management strategy, and to ensure that new management designs are economically viable.

## Introduction

Agriculture is one of many domains where a (r)evolution is essential to meet the challenge of pressing and variable environmental constraints, as well as increasing demographic pressure. Several paradigms initiated the transformation of our food and agricultural systems, such as agroecology, which proposes to apply ecological principles in agriculture^1^. Most changes are driven by the need to efficiently cope with the constraints surrounding agricultural activities: resources are finite and all ecosystems are dynamic and interconnected. Since the green revolution initiated in the 1960s, research in agriculture has been compartmentalized, with the dominant idea that there exists a human-made solution to every environmental constraint, mainly relying on the use of synthetic molecules, the artificialization of the production environments, and with plant and animal breeding goals focused only on production performances. Unfortunately, the broad impact of these solutions on ecosystem health, including the food systems and human health, has been largely underestimated^2^. Indirectly, this eradication strategy created a new set of constraints, probably stronger^3–5^. Fundamentally, the paradigm shifting toward the development of control methods that aim to reach favorable equilibrium for agricultural production is a necessity. Productivity could not be the only variable while designing new agricultural practices; ecosystems, animal, and human health must be considered too, requiring collaborative, transdisciplinary, and intersectoral approaches in accordance with the One Health concept^6,7^.

Gastro-Intestinal Nematodes (GIN) are a major threat for ruminants at pasture worldwide^8^. It is a meaningful example of how the management strategies shift from total eradication based on anthelmintic (AH) chemical treatments to management of the GIN populations in order to maintain a sustainable equilibrium favorable for animal production^9^. The 1960-1980s was the golden era for AH treatments^10^ and other strategies were ignored. Necessarily, the systematic use of AH created a selection pressure driving the apparition of AH resistant GIN strains worldwide. Nowadays, the rise of AH resistance is one major concern of small ruminant husbandry^8,11^. It is now accepted by most parasitologists that integrated GIN management is the only sustainable solution^9,12–15^, however clear and general guidelines are still needed, to change management in practice, which still often relies on systematic AH use.

Formally, the current challenge for health management in agriculture is to consider a set of broader, multidisciplinary objectives and constraints (e.g. ecosystem, animal and human health, biodiversity, productivity), under great uncertainty (e.g. climate change, availability of curative treatments and resources). Rationalizing decisions, with the available knowledge and expertise, could help in reaching this goal^16^.

In this article, we present a mathematical model to balance the impact of prevention and control in disease management. This simple framework allows to highlight and discuss the key parameters of a disease management problem, making it particularly useful for engaging stakeholders with diverse backgrounds. The model facilitates the classification of potential management actions and explores their relationships to the disease from an economic perspective. The optimality results guide decision-making by identifying which actions are worth implementing, the ideal balance between prevention and control, the required level of efficacy, and the associated costs. It is suited to support generic discussions about a disease management problem, at a global scale. Although it is a small step considering the challenge of health management listed earlier, generic frameworks are needed to help the adoption of alternatives and more integrated methods in practice.

This framework differs from previous studies in its capacity to incorporate simultaneously the effects of prevention and control strategies, allowing discussion of the optimal trade-off between both. It is illustrated in a practical case, the management of gastrointestinal nematodes, a worldwide sanitary constraint for small ruminants, where more integrated management is needed to prevent drug resistance. The framework is used to discuss the economic feasibility of alternative methods, to help in their use and development in practice.

### Related works

The use of mathematical models to optimize decisions is well-suited to various areas of animal and human health management^16,18–20^, as well as ecosystem management^21–23^. Quantifying the impact of prevention is often challenging because the outcomes of preventive actions are not always straightforward to estimate^20^.

In human health, cost-effectiveness or cost-utility ratios are commonly used. These metrics represent the cost of a preventive action divided by an estimate of health outcomes, such as quality-adjusted life years (QALYs)^24,25^, which takes into account both life expectancy and quality of life. However, this approach is difficult to transfer to the animal health context, where quantitative metrics for animal welfare remain underdeveloped. Nevertheless, the underlying principle remains the same: using data to inform decisions by balancing the relative benefits of management actions against their costs.

The use of mathematical models for disease-control decision-making has been reviewed in^16^ for livestock or more recently in^17,32^ for human health. In the former, they classified quantitative modeling into two categories: statistical and/or epidemiological models, and economic models. Economic models are further divided into five categories: (i) mathematical programming, (ii) network analysis, (iii) decision analysis, (iv) simulation, and (v) cost-benefit analysis. Most modeling works on disease management strategies fall under the fourth category, simulation models^27–29^. In these cases, decisions are not optimized per se; rather, the objective is to estimate the potential effects and utility of a given management strategy. A model of disease dynamics is first developed, and then the costs and benefits of the strategy are estimated by running the model under the proposed management scenario. This approach is particularly suitable for evaluating whether a management strategy, such as vaccination, is worthwhile^28^, but it does not necessarily identify the optimal strategy among all possible alternatives.

The work presented in this article belongs to the fifth category^30–32^, as it frames the challenge of identifying an optimal disease-control strategy as a resource allocation problem. Utility is based on estimates of the benefits and costs associated with the management strategy. We followed the fundamental steps recommended for developing quantitative models to aid decision-making^16,26^. Our process involved developing a conceptual model, formulating a mathematical representation of the decision problem, and designing an algorithmic procedure to optimize the decision. It is an extension of the works presented in^31,33^, with the particular case of a unique population and where we explicitly propose a utility function in the case of gastrointestinal nematodes in small ruminants. A major difference also comes from the fact that we both include prevention and control simultaneously, unlike in the original work, where only prevention was included.

Our approach is particularly innovative in the field of animal health management, as it focuses on identifying an optimal disease management strategy through an optimization procedure. This approach is more common in health economic^34,35^ and ecosystem management/quantitative ecology, where mathematical modeling and optimization have been widely used to address issues such as managing invasive species or conserving threatened populations^21–23^. This work draws particular inspiration from^21^, where the objective was to determine an invasive species surveillance strategy, while minimizing management costs.

### Framing a disease management problem

Mathematical models offer a valuable tool for analyzing the impact of decisions on dynamic systems. Here we provide a simple framework capturing the key elements of a disease affecting a farming production system. Management strategies are compared in terms of farmer’s profit, denoted *G*, a common variable to compare the effect of management strategies. For examples, *G* could be the gain after selling an animal product (e.g. meat, milk, eggs) or after harvesting the crop field. It could also be based on a biodiversity or a durability index, or on ecosystem services in other applications. One constraint is to be able to compare *G* with the management effort, and thus need to be on the same currency, which could be difficult. *G* is a random variable, meaning that at the start of production, its value is unknown and will only be determined at the end when the production is sold.

It is however useful to discuss management decisions before implementing them, considering the expected value of *G*, based on the chosen strategy. In essence, defining a probability distribution for the variable *G* allows for a more informed decision-making process.

We considered a generic situation applicable to both plant and animal production system. We considered two scenarios, depending either a sanitary problem occurred or not, with a probability $p_m$. When no sanitary problem occurred, with probability $1-p_m$, the farmer’s gain is optimal, denoted $G^*$, indicating that production remains unaffected by the sanitary problem. When stress occurs, with a probability of $p_m$, some losses are expected, i.e. $G<G^*$, resulting in a final gain lower than the optimal.

Two scenarios are considered after the occurence of the health issue. Firstly, production may cease entirely, resulting in complete loss. This occurs, for example, when an animal dies. Let $p_s$ denote the survival probability, indicating the likelihood of the animal or production system surviving the health stress. Thus, with a probability of $1-p_s$, $G=0$, signifying no gain is derived from the production system.

Secondly, the production system may not cease completely but rather be affected by the health stress. Consequently, only a proportion $p_r$ of the optimal gain will be obtained from the production.

To summarize, the variable *G* is equal to $G^*$, with probability $1-p_m$, is equal to $p_r G^*$ with probability $p_m p_s$, and to 0 with probability $p_m (1-p_s)$.

**Fig. 1:**
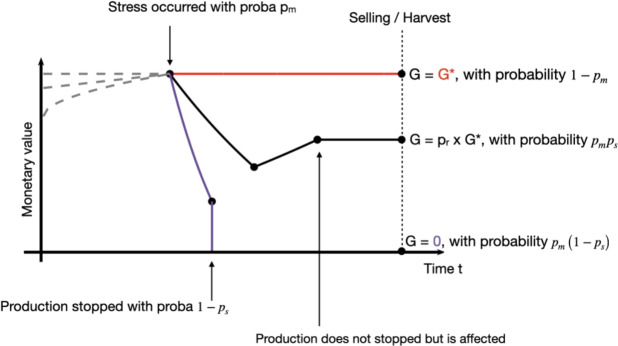
Illustration of the disease management framework: possible trajectories of the monetary value (MV) of the system over time. In normal situation, MV is an increasing function, as illustrated by the gray dotted lines. In a context of animal sold for meet for example, the monetary value mostly depends on the animal weight, which increases with time. Three scenarios are considered: (red) no sanitary stress occurred, with probability $1-p_m$ and the optimal gain $G^*$ is received. (black) the stress occurred, the production is affected, but did not stopped, with probability $p_s$, and only $p_r G$ is received. (purple) the stress occurred and totally stopped the production, no gain are received. For this last two scenarios, it can be for example the case of a fruit tree. A disease happened and some fruits are lost (decreasing monetary value). The farmer then applied some treatment. The purple line indicates a case where the treatment does not work, all the fruits are lost. The black line is another possible scenario, the treatment worked, although the lost fruits will not be sold and thus represent losses (i.e. $G<G^*$), some other fruits developed (the line slightly increased after treatment), and not all fruits were lost ($G>0$). In this illustration, the monetary value slightly increase after decreasing, for example due to posterior production of fruits. But this might not necessarily be the case, and the black line might stay constant after decreasing. This does not affect the framework, as far as this is just the value $p_r G*$ that matters. Note that for simplicity, we considered that the monetary value already reached its maximum, at the time of stress occurrence, but this might not be the case, and again, only the final values matter in the framework, not the entire trajectory.

This framework aims to describe the disease management problem using only four variables: the optimal gain $G^*$, the frequency of stress occurrence $p_m$, the survival probability of the production system in the event of a health stress $p_s$, and the system’s recovery capacity after the stress occurs $p_r$. The framework is illustrated in Fig. 1.

### Management strategy

The decision problem involves examining various management options and their impact on the final gain *G*.

Generally, there are two classes of management options: prevention and control. Prevention encompasses any management strategies implemented at the beginning of the production system, regardless of whether a health stress occurs or not. On the other hand, control strategies are only implemented upon detecting a stress.

Initially, control may seem like the optimal option because investments are only made in response to a health stress. Conversely, investments in prevention are lost if no stress occurs. This article aims to mathematically derive the situations in which it is optimal to use one or both of these management options and to discuss the practical implications of these decisions.

Prevention encompasses actions aimed at limiting the occurrence of health stress, thereby reducing $p_m$. It also includes measures to detect stress as early as possible, thereby increasing the survival probability $p_s$ or enhancing the recovery capacity $p_r$. Control, on the other hand, does not affect the frequency of stress occurrence, but only $p_s$ and $p_r$.

To compare management options, it is essential to define a criterion. A natural criterion is based on the final gain *G*, and we propose to use the expected loss due to the health stress, denoted as $V_0$:

1 $$\begin{aligned} V_0= & \textbf{E}\left[ G^*-G\right] =p_m\times \left( G^*\left( 1-p_r*p_s\right) \right) ,\nonumber \\= & p_m\times p_f. \end{aligned}$$

$\textbf{E}$ is the notation for the mathematical expectation of a random variable. $V_0$ represents the anticipated loss resulting from stress when no management action is taken, or in other words, it’s the expected disparity between the optimal benefit $G^*$ and the farmer’s actual benefit *G*. $V_0$ is the initial value of the management problem, and $p_f=G^*\left( 1-p_r*p_s\right)$ denotes the expected ultimate loss in case of stress occurrence.

Integrated Health Management (IHM) strategy aims to reduce $V_0$ through various means.

A management strategy is characterized by a combination of prevention and control efforts, denoted as $\left( x_p,x_c\right)$, along with their corresponding efficacy per unit effort $\left( \lambda _p,\lambda _c\right)$. The value of a management strategy is defined as:

2 $$\begin{aligned} V= & V_0\times \exp \left( -\lambda _p x_p-\lambda _c x_c\right) + x_p + p_m x_c. \end{aligned}$$

The expression $V_0\exp \left( -\lambda _p x_p-\lambda _c x_c\right)$ represents the updated expected loss resulting from the stress, considering the implementation of certain management efforts $x_p$ and $x_c$.

It’s important to note:

$$\exp \left( -\lambda _p x_p-\lambda _c x_c\right) =\exp \left( -\lambda _p x_p\right) \exp \left( -\lambda _c x_c\right) .$$

And the term $\Delta _p=\exp \left( -\lambda _p x_p\right)$ represents the expected reduction attributed to prevention, while $\Delta _c=\exp \left( -\lambda _c x_c\right)$ signifies the expected reduction attributed to control efforts. The expression $x_p+p_m x_c$ denotes the anticipated management cost, or simply, the expected loss solely due to management expenses. Since $x_c$ is associated only with the occurrence of stress, its anticipated cost is scaled by the probability of stress, $p_m$. The value *V* represents the anticipated loss post-management, encompassing both stress-related losses and management expenses. Utilizing *V* enables the comparison of different strategies, with the objective of minimizing this value.

The efficacies $\lambda _p$ and $\lambda _c$ quantify the rate at which the expected loss $V_0$ decreases for a given amount of prevention $x_p$ and control efforts $x_c$. Efficacy is used to model the fact that different strategy might not be as efficient than other. Or in other words, that for the same amount of effort, $x_c$ or $x_p$, we might not have the same loss reduction for all possible strategies. We used an exponential relationship to model diminishing marginal returns, implying that a small amount initially gives the biggest payoff in cost reduction^21^. Alternative increasing functions, such as logit or piecewise linear functions, could also be employed. However, they might introduce complexity in determining the optimal solution, although computing numerical solutions might be feasible.

### Optimal integrated health management strategy

The optimal integrated health management (IHM) strategy is the one allowing the optimal trade-off between loss reduction and management costs, i.e minimizes *V*. Then, the disease management problem can be reformulated as an optimization problem to determine the optimal IHM strategies based on the problem’s parameters.

The following rules of thumb can be derived from the computation of the optimal management strategy.

Management decisions should primarily depend on our ability to achieve measurable impacts on the situation, which is determined by the efficacy of our management actions. Efficacy refers to the relative value of the effects of our actions (decreasing $V_0$) compared to their costs.

An expensive management action that has little effect on the sanitary problem, i.e., on stress occurrence frequency $p_m$, individual survival when the stress occurs $p_s$, or recovery capacity $p_r$, is considered of low interest.

For prevention strategies, efficacy should be compared to the expected loss of the sanitary problem ($p_m \times p_f$). Prevention becomes worthwhile, i.e., it creates a positive trade-off between management cost and stress expected loss, when $\lambda _p > \frac{1}{p_m \times p_f}$.

Control efficacy should be compared to the loss when the stress occurs ($p_f$). Control measures are considered worthwhile when $\lambda _c > \frac{1}{p_f}$.

If the efficacies fall below these thresholds, it is not worth implementing any management action, as the investment does not justify the expected reward.

On the other hand, when the control efficacy exceeds $p_f$ and is higher than the prevention efficacy, control measures should always be prioritized.

If we achieve better results with the same investment using control measures, it is not justified to allocate resources to prevention efforts. To be the optimal strategy, the efficacy of prevention measures should significantly surpass that of control measures.

Expressing $\lambda _c = \lambda _p - \delta$, where $\delta > 0$ represents the difference in efficacy between prevention and control, it can be shown that $\delta$ must exceed $\left( 1-p_m\right) \lambda _p$ for prevention to be the optimal choice.

The term $\left( 1-p_m\right) \lambda _p$ approaches zero as $p_m$ approaches 1. In other words, the requirement for additional prevention efficacy relative to control decreases as the frequency of the sanitary problem increases.

**Fig. 2:**
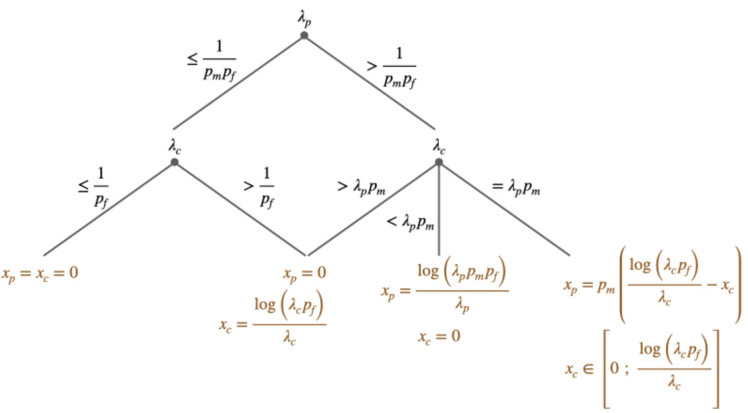
Decision tree summarizing the optimal effort allocation. Nodes represent the efficacy parameters and each branch their possible values. Final nodes are the optimal effort allocation. For example, the left part of the tree indicates that when $\lambda _p\le \frac{1}{p_m p_f}$ and $\lambda _c\le \frac{1}{p_f}$, then it is optimal to do nothing, i.e. $x^*_p=x^*_c=0$.

The optimal effort allocation is summarized in Fig. 2.

**Fig. 3:**
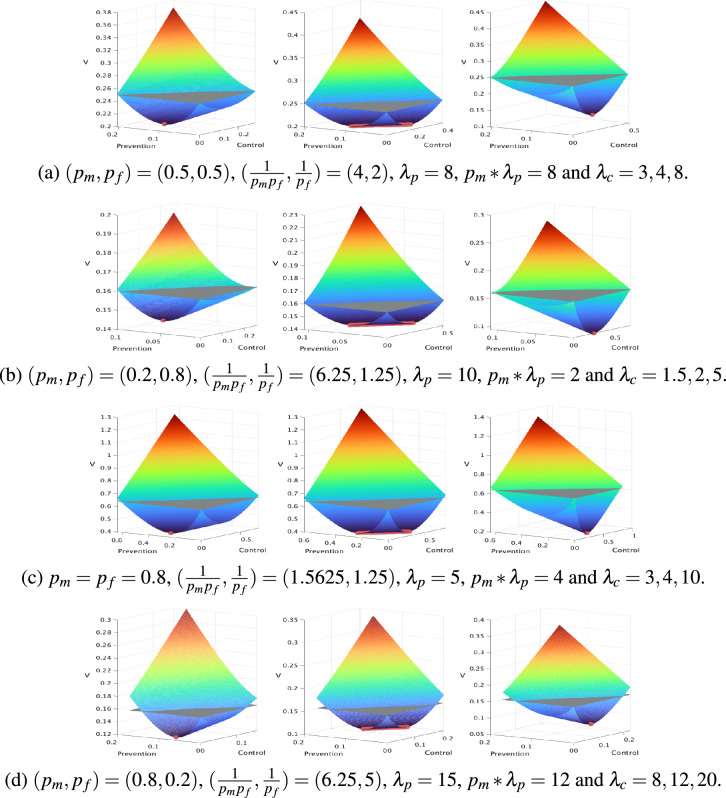
Examples of the *V* function. For each example, the function *V* is displayed in color, while the plane $z=V_0$ is displayed in gray. The *x* and *y*-axis are the amount of prevention and control efforts. Efforts are normalized with $G^*$. The *z*-axis is the value *V* of a management strategy, for the corresponding amount of prevention and control. All the parameters (prevention and control costs and *V*) are normalized by $G^*$. When *V* is under the plane, the trade-off between management effect and cost is positive. Each row, (**a**), (**b**), (**c**), (**d**) of figures gives examples when $\lambda _c > \frac{1}{p_f}$ and $\lambda _p>\frac{1}{p_m p_f}$, i.e. when a positive trade-off can be expected. Each figure gives three examples, with the same value of $p_m$, $p_f$ and $\lambda _p$, but various values of $\lambda _c$: under (left), equal (center) or above (right) $p_m*\lambda _p$. Finally, the optimal management efforts are displayed with red points on the *V* function. For each row, the left figure is for $\lambda _c<p_m*\lambda _p$. In these cases the function *V* stretch out in the prevention direction, meaning that prevention allows realizing the best trade-off between management efficacy and lost. Investing on control could also result in a positive trade-off (e.g. all the points under the plan $z=V_0$), but the optimal management strategy is obtained with no control at all. The central figures are for $\lambda _c=p_m*\lambda _p$, and in this case, there is an infinity of solutions, with bot non-zeros prevention and control efforts. The right figures are for $\lambda _c>p_m*\lambda _p$ (the opposite cases of the left figures). All the examples are obtained for $G^*=1$, a different value of *G* will just change the value on the *z* axis, but will not change the shape of the *V* function.

Several examples of the *V* function as well as optimal and sub-optimal management strategies are provided Fig. 3.

## Gastro-intestinal nematodes management in small ruminants

GIN have two main life stages: the free-living phase^39^, where they reside on the pasture ground, and the parasitic phase, where the parasites inhabit their host’s intestinal tract after being ingested during grazing.

Preventing GIN infestation primarily relies on two pillars: (i) reducing the probability of ingesting infective larvae by the host and (ii) increasing host resistance.

The first pillar, centered on pasture management, can be implemented in various ways. Rotational grazing^40^ aims to diminish the parasite load on the pasture during animal grazing. Parasites can also be removed directly from the pasture using insects^41^ or nematophagous fungi^42^, although further developments are still necessary. Another possibility is to implement a mixed-grazing system^43^ (i.e. more than one species of ruminant on the same pasture, or the same species at different physiological status). An alternative approach consists of increasing parasite mortality on pasture by either mowing or reducing irrigation. Mixed-grazing systems and rotational pasture management are the main methods investigated in recent decades.

The second pillar, based on genetic selection, nutrition, and vaccination, aims at improving the host immune response against parasites. Genetic selection^44–46^ for prevention aims at breeding resistant animals capable of ingesting or removing parasites from pasture without being impacted. Nutrition can effectively enhance host immune defenses and resistance^47–49^. A vaccine exists only for *Haemonchus contortus*^50^, which needs to be administered monthly to ensure protection. However, this solution is currently available only in South Africa and Australia.

Control also relies on two main pillars: (i) removing the parasite population within the host and (ii) increasing host resilience. The first pillar relies on anthelmintic treatments, although plants containing secondary metabolites can offer an interesting alternative^51,52^. Nowadays, it is important to reduce the use of anthelmintics to maintain their efficacy^9,13–15^. This reduction is achieved by implementing targeted selective treatments^53,54^, which involve selecting animals on an individual basis for treatment.

Finally, the second pillar relies on improved host nutrition, as well as genetic selection for resilience.

Note that resistance aims at decreasing the probability $p_m$, while resilience aims at increasing the recovery capacity $p_r$.

## Implications for GIN management in small ruminants

We illustrated our framework using the case of *Haemonchus contortus* and Creole goats under a tropical climate in Guadeloupe. Data from the PTEA INRAE experimental farm, as well as from previous studies, were utilized to estimate the model parameters.

In our flock, the pedigree of each animal is available since the foundation generation of 1979. The breeding flock consists of two sub-flocks of female Creole goats, with kids and does sharing the same pasture and being separated after weaning. All animals are reared outdoors. Kids are typically sold around 11 months of age for meat consumption. Some animals serve as substitutes for culled breeding females or male Creole Goats but are not considered in this study.

The flock of Creole bucks is smaller and reared indoors. They are allowed to graze only during the reproduction period. Breeding females alternate between two grazing areas, depending on the gestation or parturition period.

The farm collaborates with a parasitological laboratory to regularly assess the level of GIN infestation in the animals using coproscopy, particularly for the growing kids. Faecal samples are collected at 7 and 11 months of age for genetic evaluation of resistance to GIN, averaging 2 faecal egg counts (FEC) measures since 1995.

These data, along with the economic data of the farm, were used to illustrate the framework and derive the initial value of the health management problem $V_0$.

We considered the case of kids sold for meat at 11 months of age and the sanitary stress is defined as an animal with more than 500 eggs per grams of faeces. Under this threshold, the parasites population does not impact significantly animal health and welfare. The probability of an animal exceeding this threshold during its lifetime is $p_m=0.88$. After this threshold, the probability that the animal will survived without management is $p_s=0.85$, and a proportion of $p_r=0.78$ of the optimal gain will be received. The optimal gain is the average gain obtained when selling the animal (134.93 €), minus the expenses (85.39 €), $G^*=49.55$.

The initial value of the health management problem is thus $V_0=14.50$ €, which is the average loss due to parasitism. We also have $p_f=G^*(1-p_r p_s)=16.56$ €, meaning that when an animal gets infected, the expected loss if 16.65 €.

### Systematic anthelmintic treatments

In practice, control using anthelmintic treatments is the most common method and a major concern is the raise of anthelmintic resistance. We use our framework to discuss the economical impact of anthelmintic resistance. We consider that anthelmintic efficacy is related to the animal survival after infestation, but also to the loss due to the infestation when the animal survive. If $\delta _{AH}$ is the anthelmintic efficacy, we defined it as the expected proportion of the optimal gain received after infestation, or similarly that $\delta _{AH}=p_s p_r$ when anthelmintic are used. In other words, after infestation and the use of anthelmintic, $\delta _{AH} G^*$ is expected to be received. Then, when the anthelmintic is totally efficient, no loss is observed from parasitism and $\delta _{AH}=1$. In the presence of parasites resistant to anthelmintic, these parasites can survive the treatment and thus restrained growth or even lead to death.

We thus have the following relationship:

3 $$\begin{aligned} p_m G^*\left( 1-p_s p_r\right) \exp \left( -\lambda _{AH} x_{AH}\right)= & p_m G^*\left( 1-\delta _{AH}\right) \nonumber \\ \lambda _{AH}= & -\frac{1}{x_{AH}}\log \left( \frac{1-\delta _{AH}}{1-p_s p_r}\right) \end{aligned}$$

Thus, $\lambda _{AH}$ is a function of the treatment cost and AH efficacy, it is then possible to use the previous results to derive the case where AH treatment are optimal or not. We considered the common case of systematic AH treatments, i.e. animals are treated regularly, even if they are not infested. In this case, AH treatment is used as a prevention method. We accounted for 4 treatments post-weaning and 1 treatment at weaning, $x_{AH}=2.91$ €. Given the optimality results of the previous section, if systematic treatments is the only available solution, it is worth using it when $\lambda _{AH} > \frac{1}{p_m p_f}$. From rearanging Eq. (3), we can show that systematic treatment is economically viable only if the AH efficacy $\delta _{AH}>73$%. Beyond this threshold, in average, the final gain will be higher without spending money into systematic treatment.

In practice, this threshold can be used to determine whether or not to stop using an anthelmintic due to resistance. Each time animals are sold to the slaughter, farmer can compute the average weights of the animal, including the dead one, with a weight of 0. This average weight can then be compared to a theoretical one, corresponding to the weight of an healthy animal, without any problem due to parasitism. When the difference is greater than 27% then, it is not economically viable to continue using this anthelmintic.

### Targeted selective treatments

Although systematic treatment is commonly used, it is evident that it contributes to the spread of anthelmintic resistance among the parasite population. One alternative is the adoption of targeted selective treatment (TST), which involves administering anthelmintic treatment only to the infested animals. An example of this approach is the Famacha score^55^, which assesses the color of the animal’s conjunctiva to determine the level of anemia, thereby correlating it with the degree of infestation by Haemonchus. While anemia can theoretically have various causes, it is most often, and in many cases almost exclusively, attributable to parasitism, such that false positive detection could be excluded.

We model the situation of TST introducing a parameter $p_d$, the probability that the infestation is detected using a monitoring method. In the case of TST, we consider one prevention action, the monitoring method to detect infestation, and the control method, used when detected. Under the TST strategy, the probability distribution of *G* is as follows:

4 $$\begin{aligned} G = {\left\{ \begin{array}{ll} G^* & (1-p_m)\\ 0 & p_m(1-p_d)(1-p_s)\\ \delta _{AH}G^* & p_m p_d\\ p_r G^* & (1-p_d)p_s p_m\\ \end{array}\right. } \end{aligned}$$

The expected loss under the TST strategy is then equal to:

5 $$\begin{aligned} \textbf{E}\left[ G^*-G\right] =p_m G^*\left( 1-p_s p_r\right) + p_d p_m G^*\left( p_s p_r - \delta _{AH}\right) \end{aligned}$$

Similarly to Eq. (3), we can write:

6 $$\begin{aligned} p_m G^*\left( 1-p_s p_r\right) \exp \left( -\lambda _p x_p-\lambda _c x_c\right) + x_p + p_m x_c= & p_m G^*\left( 1-p_s p_r\right) + p_d p_m \left( G^* p_s p_r - G^*\delta _{AH} + x_c\right) + x_p. \end{aligned}$$

We recall that $\textbf{E}$ is the notation for the mathematical expectation of a random variable. The left side of the equation is the definition of the strategy value, using the exponential notation and the efficacy, while the right side is the value of the TST strategy in practice. Note that in the case of TST, the expected cost is $x_p + p_d p_m x_c$, as far as treatment cost occurs only if the disease is present and detected. This equality is used to derive the value of the efficacy. From the results obtained previously, using control and prevention simultaneously is optimal when $\lambda _c=p_m \lambda _p>\frac{1}{p_f}$, with an associated optimal amount of prevention and control effort (see Fig. 2). From Eq. (6), we can substitute $\lambda _c$ with $p_m \lambda _p$, and from Fig. 2, $x_p$ with:

7 $$\begin{aligned} x_p= & p_m\left( \frac{\log \left( \lambda _c p_f\right) }{\lambda _c}-x_c\right) = \frac{\log \left( \lambda _p p_m p_f\right) }{\lambda _p} -p_m x_c \end{aligned}$$

Then, we can show that:

8 $$\begin{aligned} \exp \left( -\lambda _p x_p-\lambda _c x_c\right) = \frac{1}{\lambda _p p_m p_f} \end{aligned}$$

Then Eq. (6) becomes:

9 $$\begin{aligned} \lambda _p= & \frac{1}{p_m\left( p_f+p_d\left( G^*\left( p_s p_r -\delta _{AH}\right) + x_c\right) -x_c\right) } \end{aligned}$$

Thus, we can determine the value $\lambda _p$, and, as a consequence, of $x_p$ using Eq. (7), for a given value of $p_d$, $x_c$ and $\delta _{AH}$. $x_c$ can be estimated from the farm data, as the expected control cost of the TST strategy, equals to 1.01 €.

**Fig. 4:**
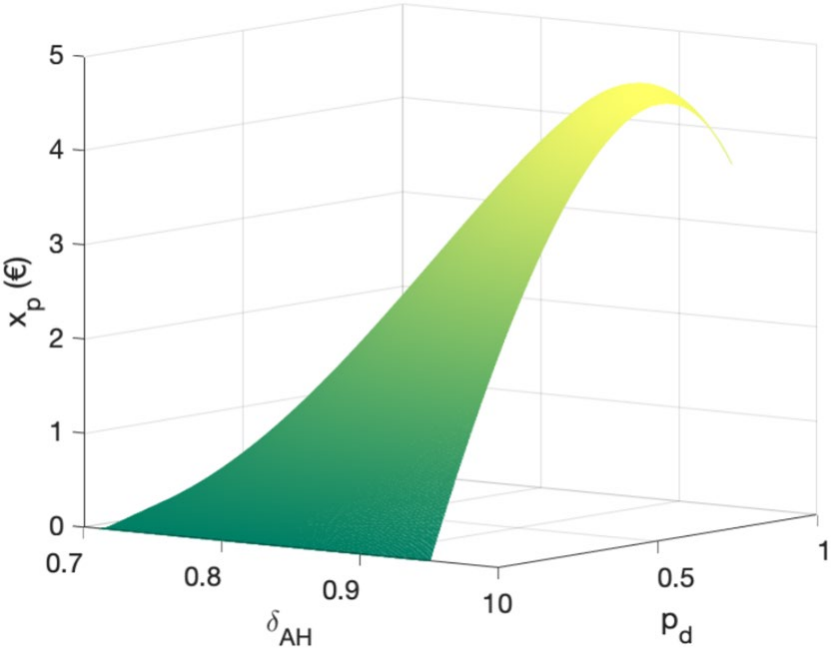
Optimal amount of monitoring ($x_p$) as a function of the probability of detecting the infestation and the efficacy of the AH treatment, under the targeted selected treatment strategy.

The value of the optimal prevention cost as a function of AH efficacy and probability of detection of the monitoring method is available Fig. (4).

As one can expect, the optimal amount of monitoring effort increases with the probability of detection and AH efficacy, until a certain threshold, where the monitoring effort slightly decrease. In this case, either the AH efficacy or probability of detection is high enough such that the expected loss due to either a failure from AH or monitoring, constraints to decrease monitoring cost. In any case, the maximal individual monitoring cost is equal to 4.45 €, for an AH efficacy of 94.2% and a probability of detection of the monitoring method of 74.6%. As an example, today, an accelerometer can be found at around 150 €, meaning that they will have to last for more than 30 years to be optimal in terms of economical value, or equivalently, used by more than 33 animals during 11 months.

### Pasture management

Finally, a common strategy consists in managing the pasture in order to limit the probability that an animal ingests parasite. It is the case for example with mixed or rotational grazing. This type of management corresponds to prevention method and we can derive the optimal economical value of prevention in the case of GIN.

Pasture management decreases the probability that an animal gets infested, or in other decreases $p_m$. Let’s denote $p_a$ the new probability that an animal gets infested under a pasture management strategy.

The expected loss under the pasture management strategy is equal to:

10 $$\begin{aligned} \textbf{E}\left[ G^*-G\right] =p_a G^*\left( 1-p_s p_r\right) \end{aligned}$$

As for Eq. (3), we can write

11 $$\begin{aligned} p_m G^*\left( 1-p_s p_r\right) \exp \left( -\lambda _p x_p-\lambda _c x_c\right) + x_p= & p_a G^*\left( 1-p_s p_r\right) + x_p. \end{aligned}$$

By rearranging Eq. (11), we can derive a value for the pasture management efficacy:

12 $$\begin{aligned} \lambda _p= & -\frac{\log \left( \frac{p_a}{p_m}\right) }{x_p} \end{aligned}$$

Using the optimality results in Fig. (2), the pasture management strategy is economically viable only if $\lambda _p> \frac{1}{p_m p_f}$, which can be written, using Eq. (12):

13 $$\begin{aligned} x_p< & -p_m p_f \log \left( \frac{p_a}{p_m}\right) \end{aligned}$$

**Fig. 5:**
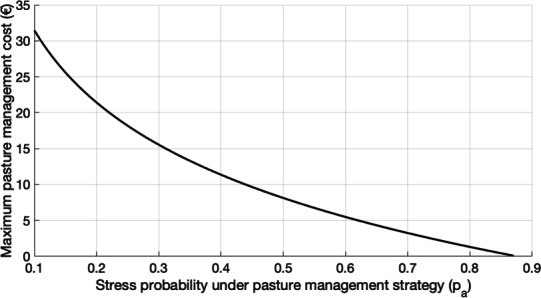
Optimal pasture management effort, in euros, as a function of the probability of getting infested under the pasture management strategy.

Figure (5) show the value of the maximal pasture management effort, as a function of the probability of getting infested under the pasture management strategy. For illustrative purpose, we used the work from^59^, which discuss the effect of stocking rate on the GIN infection levels. In this study, goats were associated to heifer that are naturally resistant to GIN. As a consequence, heifer decreases the parasite population without any impact on their health and welfare. Animal grazed in a leader goat and follower heifer design. Different design were tested, depending on the number of goats and associated heifers, insuring, however, a constant overall stocking rate between each design. In particular, they showed that the level of infection decrease with the goat stocking rate. For illustration we used the data for the design with half goats and half heifers and with 1/4 goats and 3/4 heifers. In theses cases, we can show that the probability of getting infected is respectively $p_a=0.41$ and $p_a=0.52$. Using Fig. (5), mixed grazing with 50/50 goats and heifer is economically viable when the individual cost is lower than 7.56 €. And for the 1/4 case, lower than 11.01 €.

## Discussion

Health management is a complex decision problem, where the decisions are made at multiple scales, from the country, which define legislative constraints, to the farmer, who is choosing his own health management practices. Our simplified framework aims at discussing possible improvement of health management practices, by accounting for the trade-off between reducing loss due to health problem and management cost. It can be used to guide the design of new management actions, by allowing to explore the space for which action cost and reduction loss leads to a positive trade-off. It could be particularly useful to explore and visualize the potential of prevention actions, that might be hardly used in practice as far as their impact is not necessarily easily visible. Finally, it can be used to determine futur research program effort.

The simplicity of the mathematical representation of the health management problem is particularly useful to highlight the important parameters to account for when discussing IHM strategies. First, what is the probability $p_m$ that a stress occurs and when it occurs, what are the expected losses? Second, what are the available management options, for both prevention and control? If management actions exist, what are their cost and how much losses are expected when they are implemented? Then, using this framework, one can easily illustrate the trade-off between using this action or doing nothing. If no management action exists and one wants to design a new one, then the framework could be used to explore the management cost and efficacy range for which a positive trade-off between loss reduction and management cost could be expected.

Our framework could be extended to account for environmental and human health constraints. More generally, *G* could be any quantity of interest, as far as it can be scaled with the management costs. Future developments should include temporality, especially related to the efficacy of the management actions, which could decrease with the number of times they are implemented, as it is for example the case for anthelmintic resistance.

To the best of our knowledge, this is the first mathematical model specifically designed to balance prevention and control efforts in animal health management. Although our mathematical formulations extend previous models^33^, direct comparisons remain challenging. In^31^, which is also an extension of^33^, they conclude that prevention is worthwhile for diseases with higher probability of occurrence or larger severity. Here, they did not include the possibility of choosing between prevention and control, a common decision in livestock management. It is important to consider the effects of control when discussing the use of prevention, as some diseases could have high severity, but be cured with high success.

The mathematical formulations are deliberately simple, yet they offer the significant advantage of enabling the computation of mathematically optimal management strategies. This is particularly valuable for informing decision-makers. The similarity between our framework and the concept of dynamic resilience^37,38^ is noteworthy. The black line in Fig. (1) is often employed to illustrate this concept. Furthermore,^36^ suggested using resilience as a universal health criterion applicable across various domains, such as the health of animals, soils, and humans. From this perspective, the model presented here offers potential for comparing the health, or resilience, of different agricultural systems. Historical data could be utilized to estimate disease parameters (e.g. $p_m$, $p_s$, etc), possibly integrating data from multiple diseases to account for all sanitary stress factors collectively. This approach enables a comparative analysis of system parameters to identify key differences and suggest potential improvements in resilience and overall health. Additionally, the optimality results derived from the model can inform strategies to optimize system management, thereby enhancing resilience and promoting global health outcomes.

Within the context of gastrointestinal nematodes (GIN), the question of optimal management is rarely addressed through mathematical modeling. Existing models, such as^61^ for the dynamics of GIN infestations or^62^ for the dynamics of the free-living stages, are primarily simulation models. These models have the potential to inform management decisions through further analysis and could be used as simulation tools to compare various management strategies. However, deriving mathematically optimal “rules of thumb” from these models is likely infeasible. On the contrary, we illustrated how to use this model in the context of GIN management. We first proposed a new guideline to assess whether it is economically viable to continue using an anthelmintic or if it would be better to discontinue its use due to high levels of resistance, based on animal weights. Second, we showed how to use the model to determine the maximal price of a management action. This is particularly useful, for example in the context of development of automatic monitoring method, such as accelerometers. The maximal per animal price (4.45 €  in the example) can be used as a constraint when developing this type of method, or any of them. Note that the numerical values described in this study were computed for the case of the experimental farm only. Numerical values of the health management problem and estimated costs, would be different under other context, and these parameters would have to be re-estimated to deal with a new farm/environment. However, the several examples and estimation method describe in this article can easily be generalized to other farm.

It is rare that studies abord the problem of prevention vs control, it is more often, does prevention is worth it? But not balance between prevention and control. However, this is an interesting question as far as the availability, efficacy, and cost of a control method can change the decision of using prevention or not. This was actually the case with anthelmintics, which were a low-cost and highly efficient method at the beginning. With anthelmintic resistance, this paradigm has to be changed by discussing the potential effect of alternative methods.

## Methods

### Computation of the optimal solution

Let us recall that *V* is the value of a management strategy:

$$\begin{aligned} V(x_p,x_c)= & p_m p_f \exp (-\lambda _p x_p -\lambda _c x_c) + x_p + p_m x_c.\\ \end{aligned}$$

Where $x_p$ is the prevention effort, $\lambda _p$ the prevention efficacy. And $x_c$ the control effort, $\lambda _c$ the control efficacy.

The optimal management strategy minimizes *V*, with $x_p, x_c \ge 0$, which translates to:

$$\begin{aligned} {\left\{ \begin{array}{ll} \min _{x_p,x_c}V(x_p,x_c)& \text {such that:}\\ g_p\left( x_p,x_c\right) = -x_p \le 0,& \\ g_c\left( x_p,x_c\right) = -x_c \le 0.& \\ \end{array}\right. } \end{aligned}$$

The associated Lagrangian is:

$$\begin{aligned} L(x_p,x_c,v_p,v_c)= & p_m p_f \exp (-\lambda _p x_p -\lambda _c x_c) + x_p + p_m x_c +v_p g_p\left( x_p,x_c\right) +v_c g_c\left( x_p,x_c\right) , \end{aligned}$$

The Kuhn-Tucker conditions of the optimization problems could be written as:

$$\begin{aligned} {\left\{ \begin{array}{ll} \frac{\partial L}{\partial x_p}=-\lambda _p p_m p_f \exp (-\lambda _p x_p -\lambda _c x_c) + 1 -v_p=0 & (C.1)\\ \frac{\partial L}{\partial x_c}= -\lambda _c p_m p_f \exp (-\lambda _p x_p -\lambda _c x_c) + p_m -v_c=0& (C.2)\\ v_p\ge 0& (C.3)\\ v_c\ge 0& (C.4)\\ v_p g_p\left( x_p,x_c\right) =-v_p x_p=0& (C.5)\\ v_c g_c\left( x_p,x_c\right) =-v_c x_c=0& (C.6)\\ \end{array}\right. } \end{aligned}$$

According to conditions (C.5) and (C.6), either $x_p$ or $v_p$ equal to zeros and either $x_c$ or $v_c$ are equal to zeros.

We will explore each possible scenario.

1. $$v_p=v_c=0, x_p,\ x_c\ge 0$$

In this case, according to (C.1) and (C.2), we have :

$$\begin{aligned} & -\lambda _p p_m p_f \exp (-\lambda _p x_p -\lambda _c x_c) =-\lambda _c p_f \exp (-\lambda _p x_p -\lambda _c x_c),\\ & \quad \quad \Leftrightarrow \boxed {\lambda _c=p_m\lambda _p}.\\ \end{aligned}$$

Condition (C.1) gives:

$$\begin{aligned} & \lambda _p p_m p_f \exp (-\lambda _p x_p -\lambda _c x_c) =1\\ & \quad \Leftrightarrow \exp (-\lambda _p x_p -\lambda _c x_c)=\frac{1}{\lambda _p p_m p_f }\\ & \quad \Leftrightarrow \lambda _p x_p + \lambda _c x_c=\log \left( \lambda _p p_m p_f \right) \\ & \quad \Leftrightarrow x_p =\frac{\log \left( \lambda _p p_m p_f \right) - \lambda _c x_c}{\lambda _p }\\ & \quad \Leftrightarrow x_p =\frac{\log \left( \lambda _c p_f \right) - \lambda _c x_c}{\frac{\lambda _c}{p_m} }\\ & \quad \Leftrightarrow \boxed {x_p =p_m\left( \frac{\log \left( \lambda _c p_f \right) }{\lambda _c }- x_c\right) }\\ \end{aligned}$$

To guaranty that $x_p\ge 0$, we need:

$$\begin{aligned} & \log \left( \lambda _c p_f \right) - \lambda _c x_c \ge 0,\\ & \quad \Leftrightarrow x_c \le \frac{\log \left( \lambda _c p_f \right) }{\lambda _c},\\ & \quad \Leftrightarrow x_c \in \left[ 0\;\ \frac{\log \left( \lambda _c p_f \right) }{\lambda _c}\right] \\ \end{aligned}$$

For this first scenario, there exists an infinity of solutions.

2 $$v_p=0, v_c >0, x_p\ge 0, x_c =0$$

We have:

$$\begin{aligned} & \frac{\partial L}{\partial x_p}=-\lambda _p p_m p_f \exp (-\lambda _p x_p) + 1=0,\\ & \quad \Leftrightarrow \boxed {x_p =\frac{\log \left( \lambda _p p_m p_f\right) }{\lambda _p}.}\\ \end{aligned}$$

To guaranty that $x_p\ge 0$, we need:

$$\begin{aligned} & x_p\ge 0,\\ & \quad \Leftrightarrow \log \left( \lambda _p p_m p_f\right) \ge 0,\\ & \quad \Leftrightarrow \lambda _p p_m p_f \ge 1,\\ & \quad \Leftrightarrow \boxed {\lambda _p \ge \frac{1}{p_m p_f}.}\\ \end{aligned}$$

We can then deduce the value of $v_c$ :

$$\begin{aligned} \frac{\partial L}{\partial x_c}=0\Leftrightarrow & -\lambda _c p_m p_f\exp (-\lambda _p x_p)+p_m-v_c =0 \\ \end{aligned}$$

We substitute the value of $x_p$ obtained previously, we have:

$$\begin{aligned} \frac{\partial L}{\partial x_c}=0\Leftrightarrow & -\lambda _c p_m p_f\frac{1}{\lambda _p p_m p_f}+p_m-v_c =0 \\\Leftrightarrow & \underline{v_c =p_m -\frac{\lambda _c}{\lambda _p}} \\ \end{aligned}$$

Finally, the strict positivity of $v_c$ implies:

$$\begin{aligned} v_c > 0\Leftrightarrow & \frac{\lambda _c}{\lambda _p}< p_m \\\Leftrightarrow & \boxed {\lambda _c < \lambda _p p_m} \\ \end{aligned}$$

3 $$v_p > 0, v_c =0, x_c\ge 0, x_p =0$$

We have:

$$\begin{aligned} & \frac{\partial L}{\partial x_c}=-\lambda _c p_m p_f \exp (-\lambda _c x_c) + p_m=0,\\ & \quad \Leftrightarrow \boxed {x_c =\frac{\log \left( \lambda _c p_f\right) }{\lambda _c}.}\\ \end{aligned}$$

To guaranty that $x_c\ge 0$, we need:

$$\begin{aligned} & x_c\ge 0,\\ & \quad \Leftrightarrow \log \left( \lambda _c p_f\right) \ge 0,\\ & \quad \Leftrightarrow \lambda _c p_f \ge 1,\\ & \quad \Leftrightarrow \boxed {\lambda _c \ge \frac{1}{p_f}.}\\ \end{aligned}$$

From which we can deduce the value of $v_p$ :

$$\begin{aligned} \frac{\partial L}{\partial x_p}=0\Leftrightarrow & -\lambda _p p_m p_f\exp (-\lambda _c x_c)+1-v_p =0 \\ \end{aligned}$$

We substitute the value of $x_c$ obtained previously, we have:

$$\begin{aligned} \frac{\partial L}{\partial x_p}=0\Leftrightarrow & -\lambda _p p_m p_f\frac{1}{\lambda _c p_f}+1-v_p =0 \\\Leftrightarrow & \underline{v_p =1 -\frac{\lambda _p}{\lambda _c}}p_m \\ \end{aligned}$$

Finally, the strict positivity of $v_p$ implies:

$$\begin{aligned} v_p> 0\Leftrightarrow & \frac{\lambda _p}{\lambda _c}p_m < 1\\\Leftrightarrow & \boxed {\lambda _c > \lambda _p p_m} \\ \end{aligned}$$

4 $$v_p>0, v_c >0, x_p= x_c =0$$

In this case, the solution is simply $\boxed {x_p= x_c =0}$.

We can first deduce the value of $v_p$ :

$$\begin{aligned} \frac{\partial L}{\partial x_p}=0\Leftrightarrow & -\lambda _p p_m p_f+1-v_p =0, \\\Leftrightarrow & \underline{v_p = 1-\lambda _p p_m p_f}.\\ \end{aligned}$$

The strict positivity of $v_p$ implies that:

$$\begin{aligned} v_p > 0\Leftrightarrow & \lambda _p p_m p_f< 1,\\\Leftrightarrow & \boxed {\lambda _p < \frac{1}{p_m p_f}.} \\ \end{aligned}$$

We can deduce the value of $v_c$ :

$$\begin{aligned} \frac{\partial L}{\partial x_c}=0\Leftrightarrow & -\lambda _c p_m p_f+p_m-v_c =0 \\\Leftrightarrow & v_c = p_m\left( 1-\lambda _c p_f\right) \end{aligned}$$

And the strict positivity of $v_c$ implies that:

$$\begin{aligned} v_c > 0\Leftrightarrow & \lambda _c p_f<1 \\\Leftrightarrow & \boxed {\lambda _c <\frac{1}{p_f}} \\ \end{aligned}$$

### Parameter estimation of the health management problem in the case of gastro-intestinal management under tropical conditions

We first start with the estimation of the health management problem parameters. We mainly relied on the work presented in^57^, where they studied two scenarios, one where the animals are treated in a systematic way, 4 times during their lifetime, with effective AH treatments. And a second scenario, where no anthelmintic treatments were used. Thus, in scenario 1, the production value are in a case where parasitism is not a problem and where $G^*$ is reached at the end of the animal lifetime. On the contrary, in scenario 2, it is expected that production could be lower, due to parasitism. By comparing this two scenarios, we were able to derive most of our model parameters.

Starting with the mortality. In the first scenario, pre and post weaning mortality were 18% and 12%. The combined mortality is thus equal to $12 + 0.18*(100-12)=27.84\%$. In the second scenario, pre and post weaning mortality were 29% and 19%. In this case, the combined mortality is equal to $29 +.19*(100-29)=42.46\%$ The difference in mortality, $42.46\% - 27.84\%=14.65\%$, is due to parasitism. In other words, we have $1-p_s=14.65\%$, or $p_s=0.8535$.

For the estimation of the optimal gain, we updated the data from the reference article^57^ with the economical data of the farm, computed in 2024. Otherwise, the economical conclusion of our article would be outdated. We used a carcasse price of kids at 11 months of 7.70 €, instead of 11.96 €  in the reference article. In scenario 1, the average carcasse weight at 11 months was equal to 17.9kg, then the average price was $17.9\times 7.70=137.83$ €. We deducted national taxes, at 2.10%, to estimate the average income to 134.93 €. In scenario 2, the average carcasse weight at 11 months was down to 13.9kg, consequently with an average price of $(7.70\times 13.9)\times 97.9\%=104.78$ €. Then, we can estimate the proportion loss due to parasitisme $p_r=\frac{104.78}{134.9}=0.78$. The expense were estimated as follows. First, we accounted for a management cost of 83.52 €  for the adults. It includes 39.32 €  for hay, 36.48 €  for concentrate, 2.43 €  for treatment against ticks and 5.29 €  for GIN. We accounted for an average litter size of 2.3, and thus, the management cost for the dam is $\frac{83.52}{2.3}=36.31$ €. For the kids, we accounted for 20.76 €  for hay, 14.61 €  for concentrate, 2.43 €  for ticks treatment, 7.75 €  for Coccidies, 1.53 €  for Taenia, and, 2 €  for the animal national identification. In total, the production costs was estimated to $36.31+49.08=85.39$ €  and thus $G^*=134.93-85.39=49.55$ €.

For the estimation of $p_m$, the probability that an animal gets infested by the parasitism, we used the following method. First we used a dataset containing the results of 245 coproscopies, made on 129 different animals in 2022-2023. A coproscopie is a method used to determine the level of infestation of the animals. More precisely, to estimate the number of GIN eggs per gram (EPG) of feces. Theoretically, an animal is infected as soon as its EPG is higher than 0. But in practice, we considered that animals with EPG $\le 500$, were healthy, as far as there is no impact on their health and welfare. The coproscopies were realized at 4 or 7 months of age. They were realized for genetic evaluation of the animal’s level of genetic resistance to GIN. This dataset is not publish at this time. The EPG of the animals was estimated multiple time, spaced from 5, 7 or 9 days. For each animal, we used the average value of the estimated EPG. To summarize, we have the infestation level (EPG) of 129 animal, at 4 or 7 months of age. We used this dataset to answer this first question: what is the probability that an animal that a raised on pasture is infected (i.e. EPG > 500) at a given day? This probability is simply the number of infected animal in our dataset, divided by the number of animals in the dataset. It is equal to 0.34. In other word, in the experimental farm, if we choose an animal randomly, for a given day, there is 34% chance that this animal is infected. Note that the coproscopies were realized at different periods of the year, in January, May, or September, thus with various climate conditions (more or less dry). But also with animals from various levels of genetic resistance to the parasites. The previous probability is an estimate with average climatic conditions and genetic backgrounds.

Then, we asked the following question: if an animal is infected at a given time (i.e. EPG > 500), when did the animal ingest larvae? We used the work from^60^, where 11 months old Creole kids were infected experimentally with a daily dose of 1000 larvae of *Haemonchus contortus* during 10 days. Trickle infections were used to better mimic natural infection condition on pasture. We used this dataset, containing 29 animals, to compute the average time where the animal get an EPG > 500, or in our definition, gets infected. The estimation showed that, in average, EPG was higher than 500 after 50.6 days. In other words, when an animal is exposed to larvae, it will be infected (EPG > 500) at least 50.6 days after. Mathematically, let’s define the following Bernoulli trial: at the end of a period of 50.6 days during the animal’s lifetime, the animal is either infected (success) or non-infected (failure). Then the probability of success is 34%.

Our aim was to compute the probability that an animal gets infected during its entire lifetime, i.e. before 11 months. The potential infection period was reduced to 9 months, as far as before 2 months of age, the animals were essentially fed with milk from the dam. We then approximately derive the lifetime of the animal into 6 periods of 50.6 days and we defined $p_m$, as the probability that the animal gets infested in at least one of this period. To compute this probability, we can delete the first period, as far as if the animal is exposed to larvae during this period, it will get infected (EPG > 500) in the next period. Then, $p_m$ can be viewed as the probability of at least one success, in a sequence of 5 Bernoulli trials, with success probability of 0.34. In this terms, $p_m$ can be easily calculated using the binomial probability, and is equal to $p_m= 0.88$.

### Systematic anthelmintic treatments

The only extra parameter to estimate was the anthelmintic treatment cost $x_{AH}$. We used the anthelmintic price at the experimental farm in 2024, and accounted for 1 treatment (0.53 €) at weaning and 4 treatments after weaning (2.38 €), for a treatment cost of $x_{AH}=0.53 + 2.38=2.91$.

### Targeted selective treatments

For the targeted selective treatments, we computed the average cost of treatment, which depends on the number of times the animal gets infested. The treatment costs was estimated to $x_c=\frac{2.38}{4}=0.595$. Then, we used the Binomial probability defined earlier, with a success probability of 0.34 to compute the expected treatment cost:

$$x_{TST}=\sum _{i=1}^5 i*x_c*Bino\left( 5-i,5,0.34\right) =1.01,$$

where $Bino\left( s,5,0.34\right)$ is the binomial probability of *s* successes for 5 Bernoulli trials with a success probability of 0.34.

## Data Availability

All data generated or analysed during this study are included in this published article.
